# Treatment-emergent major adverse cardiovascular and thromboembolic events were infrequent during clinical trials of pegloticase

**DOI:** 10.1093/rheumatology/keaf017

**Published:** 2025-01-10

**Authors:** Orrin M Troum, Mai Duong, Katie Obermeyer, Lissa Padnick-Silver, Brian LaMoreaux

**Affiliations:** Division of Rheumatology, University of Southern California Keck School of Medicine and Providence Saint John’s Health Center, Santa Monica, CA, USA; Amgen Inc., Thousand Oaks, CA, USA; Amgen Inc., Thousand Oaks, CA, USA; Amgen Inc., Thousand Oaks, CA, USA; Amgen Inc., Thousand Oaks, CA, USA

**Keywords:** gout, flare, pegloticase, cardiovascular event, venous thromboembolic event, serum urate, urate-lowering therapy, tophus, pooled analysis

## Abstract

**Objectives:**

Long-term maintenance of serum urate levels <6 mg/dl reduces gout flare frequency. However, urate-lowering therapy (ULT) initiation can induce gout flare. The incidence of thromboembolic (TE) and cardiovascular (CV) events has been shown to increase in the 30 and 120 days following gout flare, respectively; therefore, the question of ULT initiation increasing patient risk for CV/TE events has been raised. Here, we investigate CV/TE event incidence following pegloticase initiation in clinical trials.

**Methods:**

This *post hoc* analysis of pooled data from four trials examined treatment-emergent gout flare and CV/TE events in patients with uncontrolled gout. Studies included two phase 3 trials (NCT00325195), the MIRROR open-label trial (NCT03635957), and the MIRROR randomized controlled trial (NCT03994731). Per protocol, pegloticase (8 mg) was administered every 2 (all trials) or 4 weeks (phase 3 trials); data from the first 24 weeks of therapy were included in this analysis. Some MIRROR patients received MTX (15 mg/week) as co-therapy. Based on prior studies, the high-risk window for CV/TE events was defined as 120 days following flare onset.

**Results:**

Overall, 5/328 (1.5%) patients experienced ≥1 CV/TE event during pegloticase treatment, including 3/244 (1.2%) patients who received on-label (biweekly) dosing (35.4 events/1000 person-years). All events occurred within the 120-day gout flare exposure window.

**Conclusions:**

CV/TE event incidence during pegloticase treatment was similar to the general gout population (31.7 events/1000 person-years). These findings suggest that pegloticase initiation does not put patients at a higher risk for CV/TE events.

Rheumatology key messages

- Urate-lowering therapy initiation can cause flares, which may put patients at a higher risk for cardiovascular/thromboembolic events.
- This pooled trial analysis showed that cardiovascular/thromboembolic events were rare during pegloticase treatment, occurring at a similar incidence to the general gout population.
- Pegloticase clinical trials did not indicate any cardiovascular safety signals; this finding was further supported with this pooled analysis.

## Introduction

Gout is a common form of inflammatory arthritis with a prevalence of 5.1% (12.1 million people) in the US that is highly treatable but often ineffectively managed [1, 2]. The disease negatively impacts patients’ quality of life, with the number of affected joints and gout flare frequency significantly contributing to these outcomes [3–7].

Gout is associated with a high prevalence of cardiovascular (CV), metabolic and renal comorbidities [8–10], with an even higher prevalence in people with uncontrolled gout [11]. According to the US National Health and Nutrition Examination Survey, over 70% of US adults with gout had hypertension and/or chronic kidney disease, over 50% had obesity and over 10% had a myocardial infarction and/or heart failure, all representing rates up to three times higher than in those without gout [8]. Interestingly, the risks of venous thromboembolic (TE) and CV events were shown to be higher within the 30 and 120 days, respectively, after gout flare [12, 13]. This may stem from flare-related neutrophilic inflammation, which is associated with atherosclerotic plaque instability and rupture [13]. Therefore, the maintenance of serum urate (SU) levels <6 mg/dl, which reduces gout flare recurrence, could play an important role in maintaining patient quality of life and overall health [14].

Initiation of an oral urate-lowering therapy (ULT) can induce gout flares, as monosodium urate crystals mobilize from articular deposits with falling SU levels [15]. The mobilization of these crystals can induce an acute inflammatory response, and gout flare frequencies may remain elevated for up to 6 months following oral ULT initiation [14, 15]. Pegloticase, an infused pegylated uricase enzyme, can lower SU levels in patients refractory to or intolerant of oral ULTs [16]; however, gout flare was the most common treatment-emergent adverse event in clinical trials. In these trials, the proportion of patients experiencing gout flares increased during the first month of pegloticase therapy but rapidly and progressively declined to nearly 0% over 12 months of therapy [17]. Although CV safety signals were not identified in pegloticase clinical trials, the demonstrated relationship between gout flares and CV/TE events has raised the important question of whether or not pegloticase treatment increases these events in uncontrolled gout patients. Therefore, the occurrence of CV/TE events across clinical trials of pegloticase was further examined in this pooled *post hoc* analysis of clinical trial data.

## Methods

This pooled *post hoc* analysis included data from patients who received ≥1 pegloticase infusion as part of four pegloticase clinical trials. These trials included two phase 3 pivotal trials (8 mg pegloticase infusion every 2 weeks [Q2W] or 8 mg pegloticase infusion every 4 weeks [Q4W]; NCT00325195), the MIRROR open-label (MIRROR OL) trial (8 mg pegloticase infusion Q2W + 15 mg/week oral MTX; NCT03635957) and the MIRROR randomized controlled trial (MIRROR RCT; 8 mg pegloticase Q2W + 15 mg MTX weekly or 8 mg pegloticase Q2W + placebo; NCT03994731) [16, 18, 19].

### Patients

All patients had uncontrolled gout, defined as elevated SU (phase 3: ≥8 mg/dl; MIRROR OL: ≥6 mg/dl; MIRROR RCT: ≥7 mg/dl), oral ULT intolerance/inefficacy and ongoing gout signs/symptoms (≥2 flares in the prior year, ≥1 tophus and/or gouty arthropathy). All patients received standard gout flare prophylaxis for ≥1 week prior to the first pegloticase infusion in all trials. Standard pre-infusion prophylaxis was administered prior to each pegloticase dose, including 125 mg intravenous methylprednisolone or 200 mg hydrocortisone. In the MIRROR OL trial, all patients received 15 mg/week of oral MTX as co-therapy to pegloticase beginning 4 weeks prior to the first pegloticase infusion. In MIRROR RCT, patients were randomized 2:1 to receive 15 mg/week of oral MTX or placebo as co-therapy beginning 4 weeks prior to the first pegloticase infusion. All patients in MIRROR RCT also underwent a 2-week MTX tolerance assessment prior to randomization. Both MIRROR trials included up to 52 weeks of pegloticase treatment, and the phase 3 trials included up to 24 weeks of pegloticase treatment. Apart from these noted differences, protocols were similar across the four trials [16, 18, 19].

### Outcomes and assessments

Treatment-emergent events were defined as those occurring between the first pegloticase infusion (day 1) and 30 days after the last pegloticase or MTX dosing, whichever was later, in the MIRROR trials through week 24 and any time after the first dose of pegloticase in the phase 3 pivotal trials. Treatment-emergent gout flares and CV/TE events were identified and reported during the first 24 weeks of pegloticase therapy. CV/TE events included major adverse cardiovascular events (nonfatal myocardial infarction, nonfatal stroke and CV death), congestive heart failure and venous TE. The incidence of CV and TE events following day 1 was calculated, and the time between gout flare and each identified CV/TE event was assessed. Based on similar analyses reported in the literature, a gout flare exposure window of 120 days was used [12, 13].

### Statistical analysis

Analyses were based on observed data. Categorical parameters are presented as *n* (%); continuous parameters are presented as mean (s.d.) or median (range) depending on sample size. Incidence rates per 1000 patient-years were calculated as 1000 × (total number of events/total patient-years of pegloticase). The total patient-years of pegloticase exposure was calculated as Sum([day of last dose through week 24 − day of first dose + 1]/365.25). It should be noted that this analysis only examined treatment-emergent flares and does not account for flares that occurred prior to the first pegloticase infusion.

### Study approval

All four pegloticase trials were undertaken in accordance with the principles of the Declaration of Helsinki and the International Conference on Harmonisation Guidelines for Good Clinical Practice. As this was a *post hoc* analysis of anonymized data, no additional patient informed consent or ethics committee or institutional review board approvals were required. All such consents and approvals were obtained in the original trials [16, 18, 19].

## Results

### Patient characteristics

A total of 328 patients received pegloticase across all trials (Q2W dosing: *n* = 244; Q4W dosing: *n* = 84). The majority of patients were white (68.3%) and male (85.7%), with a mean (s.d.) age of 54.9 (13.5) years. Of the 244 patients who received pegloticase biweekly, 110 also received MTX as co-therapy (MIRROR OL: *n* = 14; MIRROR RCT: *n* = 96). As with the full patient set, patients receiving pegloticase Q2W were predominately white (67.6%) and male (86.9%) and had a mean (s.d.) age of 55.0 (13.5) years and BMI of 33.0 (6.8) kg/m^2^ (Table 1). On average (s.d.), patients who received pegloticase Q2W had a gout duration of 14.3 (11.0) years, an SU level of 9.3 (2.6) mg/dl and experienced 9.3 (12.2) acute gout flares (range: 0–110) in the 12 months prior to study enrolment.

**Table 1.** Patient demographics and baseline characteristics by treatment group

	Pegloticase Q2W monotherapy	Pegloticase Q2W + MTX	Pegloticase Q4W monotherapy
	*N* = 134	*N* = 110	*N* = 84
Age, years, mean (s.d.)	54.9 (14.5)	55.1 (12.3)	54.5 (13.3)
Male, *n* (%)	110 (82.1)	102 (92.7)	69 (82.1)
White, *n* (%)	87 (64.9)	78 (70.9)	59 (70.2)
BMI, kg/m^2^, mean (s.d.)	33.0 (7.5)	32.9 (5.8)	32.9 (8.0)
Number of flares over previous 12 months, mean (s.d.)	8.3 (11.9)	10.6 (12.5)	6.4 (7.5)
Baseline SU, mg/dl, mean (s.d.)	9.8 (2.0)	8.8 (1.6)	10.3 (1.8)
Patients with tophi, *n* (%)	101 (75.4)	84 (76.4)	64 (76.2)
Gout duration, years, mean (s.d.)	14.7 (11.6)	13.7 (10.3)	15.0 (9.8)
eGFR, ml/min/1.73 m^2^, mean (s.d.)	63.3 (22.9)	71.2 (18.9)	57.3 (23.4)

eGFR: estimated glomerular filtration rate; Q2W: every 2 weeks; Q4W: every 4 weeks; SU: serum urate.

### Treatment-emergent gout flares during Q2W dosing of pegloticase

On-label pegloticase treatment includes 8 mg infusions administered Q2W; therefore, flare analyses focused on patients who received Q2W dosing. At least one treatment-emergent gout flare occurred in 176/244 (72.1%) patients who received pegloticase on a Q2W dosing schedule. Patients who did (*n* = 176) vs did not (*n* = 68) experience at least one flare had similar baseline demographics and gout characteristics (Table 2), but those with a treatment-emergent flare had a higher flare rate at baseline (mean [s.d.]: 10.9 [13.6] vs 5.3 [5.7] flares in the prior 12 months; median [range]: 7 [0–110] vs 3 [0–30]).

**Table 2.** Patient demographics and baseline characteristics by treatment-emergent flare occurrence

	Pegloticase Q2W monotherapy		Pegloticase Q2W + MTX	
	*N* = 134		*N* = 110	
	Gout flares	No gout flares	Gout flares	No gout flares
	*n* = 99	*n* = 35	*n* = 77	*n* = 33
Age, years, mean (s.d.)	54.9 (14.4)	54.9 (15.0)	53.2 (12.3)	59.5 (11.3)
Male, *n* (%)	84 (84.8)	26 (74.3)	73 (94.8)	29 (87.9)
White, *n* (%)	69 (69.7)	18 (51.4)	56 (72.7)	22 (66.7)
BMI, kg/m^2^, mean (s.d.)	32.6 (7.0)	34.0 (8.9)	32.6 (5.6)	33.6 (6.2)
Number of flares over previous 12 months, mean (s.d.)	9.6 (13.3)	4.8 (5.1)	12.6 (13.9)	5.7 (6.3)
Baseline SU, mg/dl, mean (s.d.)	10.0 (1.8)	9.1 (2.3)	8.9 (1.7)	8.8 (1.6)
Patients with tophi, *n* (%)	79 (79.8)	22 (62.9)	61 (79.2)	23 (69.7)
Gout duration, years, mean (s.d.)	16 (11.9)	13 (10.4)	14.5 (10.5)	11.9 (9.9)
eGFR, ml/min/1.73 m^2^, mean (s.d.)	63.4 (23.7)	63.3 (20.8)	71.0 (18.0)	71.7 (21.3)

Flare groups are based on the occurrence of treatment-emergent gout flares, defined as occurring between the date of the first pegloticase infusion (day 1) and 30 days after the last pegloticase or MTX dosing in the MIRROR trials and after the first dose of pegloticase in the phase 3 pivotal trials.

eGFR: estimated glomerular filtration rate; Q2W: every 2 weeks; SU: serum urate.

The mean number of treatment-emergent gout flares was not captured as a part of the pooled analysis. However, this information is available for the MIRROR OL trial (all patients received MTX co-therapy) and MIRROR RCT, some of which is already published. In the MIRROR OL trial, the proportion of patients experiencing a treatment-emergent flare during months 1–3 (day 1–week 12) and months 4–6 (weeks 12–24) of treatment was 92.9% and 41.7%, respectively [20]. Among patients who experienced ≥1 treatment-emergent flare, a mean (s.d.) of 4.2 (2.3; range: 1–8) and 3.2 (2.4; range: 1–7) flares were reported during months 1–3 and months 4–6 of treatment, respectively [20]. In MIRROR RCT, the proportion of patients experiencing a treatment-emergent flare during months 1–3 was 65.6% with pegloticase + MTX vs 69.4% with pegloticase + placebo; during months 4–6 the proportion was 27.1% vs 14.3% [21]. In patients who experienced ≥1 treatment-emergent flare, the mean (s.d.) number of flares during months 1–3 was 2.6 (2.0; range: 1–9) with pegloticase + MTX vs 2.2 (1.5; range 1–6) with pegloticase + placebo; during months 4–6 the number of flares was 2.1 (1.2; range: 1–5) vs 1.9 (1.5; range 1–5). It should be noted that in MIRROR RCT, the use of MTX co-therapy had no observed influence on the frequency or severity of gout flares [17, 19]. In the phase 3 trials, the proportion of patients experiencing a treatment-emergent flare during months 1–3 and months 4–6 of pegloticase Q2W treatment was 74.1% and 40.6%, respectively [21].

### CV/TE events during pegloticase treatment

**Figure 1.**
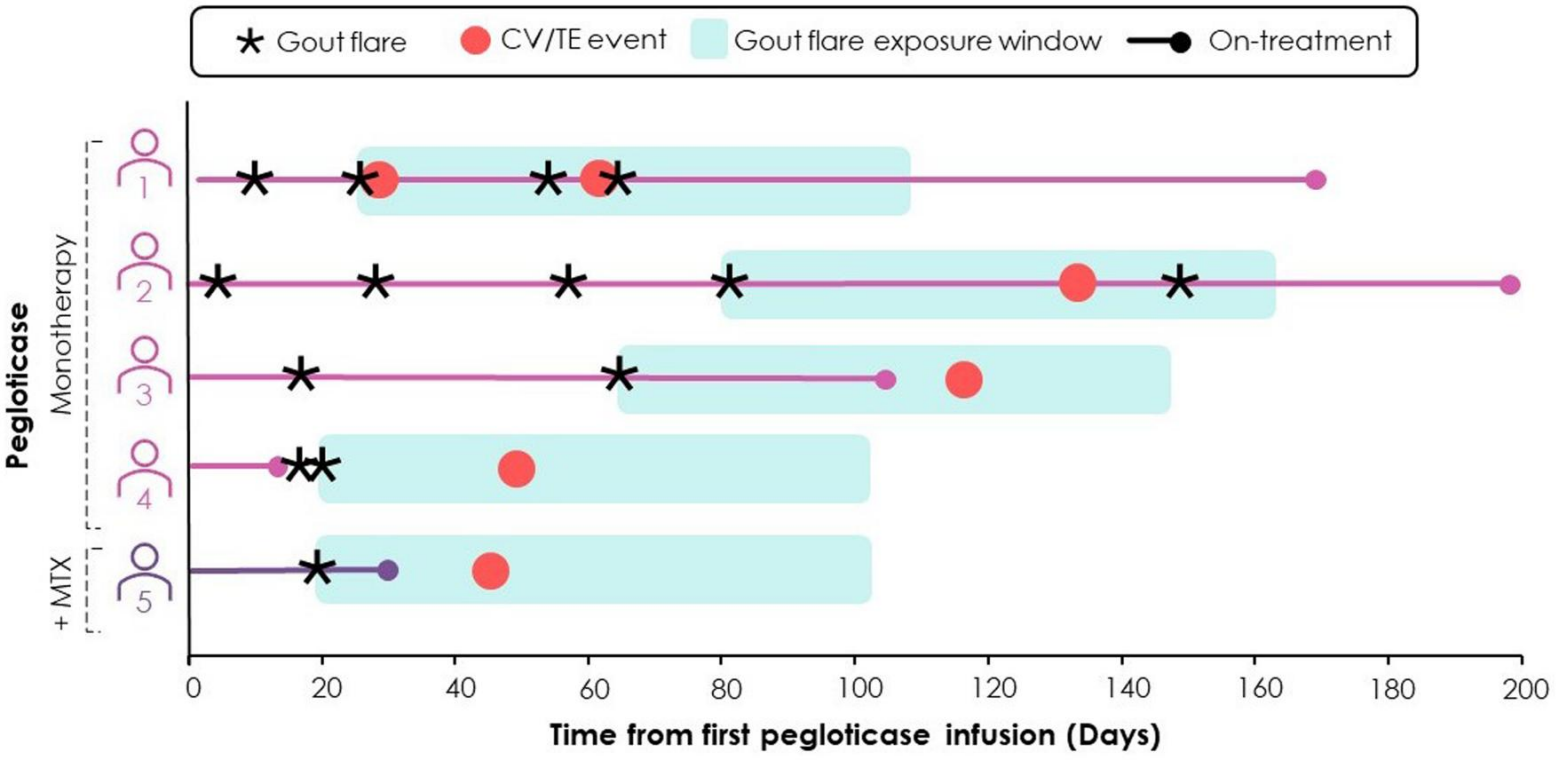
Timing of gout flare and CV/TE events relative to pegloticase initiation. Patients 1 through 4 were enrolled in the phase 3 trial (pegloticase monotherapy); patient 5 was enrolled in MIRROR RCT (pegloticase + MTX). The 120-day flare exposure window is marked from the day of a flare prior to the first CV/TE event. CV: cardiovascular; MIRROR: MTX to increase response rates in patients with uncontrolled gout receiving pegloticase; RCT: randomized controlled trial; TE: thromboembolic

Overall, 5/328 (1.5%) patients experienced ≥1 treatment-emergent CV/TE event (Table 3). Four of these patients had received pegloticase monotherapy as part of the phase 3 trial, all of whom had received colchicine gout flare prophylaxis. The other patient with a CV/TE event received pegloticase + MTX as part of MIRROR RCT and received ibuprofen for gout flare prophylaxis. All CV/TE events occurred within the 120-day gout flare exposure window (Table 4; Fig. 1). Although analyses are restricted to events through week 24 for the MIRROR trials, it should be noted that no CV/TE event occurred after week 24 in either trial. The median time from the first treatment-emergent flare to a CV/TE event was 35 days (range: 18–130 days). The median time from the most recent treatment-emergent flare to a CV/TE event was 33 days (range: 3–69 days).

**Table 3.** Characteristics of patients who experienced a CV/TE event during pegloticase treatment administered in the clinical trial setting

Patient	Age (years)	Sex	Race	BMI (kg/m^2^)	Gout history (years)	Tophi	Number of flares over the last 18 months	Comorbidities related to CV/TE risk
1	73	Male	White	32.2	32	No	10	HTN, cardiac arrhythmia, hyperkalaemia, CKD, hyperlipidaemia
2	76	Male	Black	33.6	16	Yes	8	HTN, cardiac arrhythmia, LE oedema, hyperkalaemia, CKD, renal failure
3	61	Male	White	33.3	6	Yes	15	HTN, angina, CHF, amputation, PVD, NIDDM
4	78	Female	White	38.7	7	No	5	HTN, chest pain, cardiac arrhythmia, AA calcification, hyperlipidaemia, diabetes
5	56	Male	White	28.3	10	Yes	8^a^	None

Patients 1 through 4 were enrolled in the phase 3 trials (received pegloticase monotherapy); patient 5 was enrolled in MIRROR RCT (received pegloticase + MTX).

a Number of flares over the last 12 months (18-month data not available).

AA: abdominal aorta; CHF: congestive heart failure; CKD: chronic kidney disease; CV: cardiovascular; HTN: hypertension; LE: lower extremity; MIRROR: MTX to increase response rates in patients with uncontrolled gout receiving pegloticase; NIDDM: non-insulin-dependent diabetes mellitus; PVD: peripheral vascular disease; RCT: randomized controlled trial; TE: thromboembolic.

**Table 4.** Treatment parameters of pegloticase clinical trial patients who experienced a CV/TE event during treatment

Patient	Treatment^a^	Pegloticase dosing	Flare prophylaxis	CV/TE event(s)	Within flare exposure window
1	Pegloticase monotherapy (phase 3)	Monthly	Colchicine (0.6 mg QOD)	Acute MI, DVT	Yes, Yes
2	Pegloticase monotherapy (phase 3)	Q2W	Colchicine (0.6 mg BID)	CHF	Yes
3	Pegloticase monotherapy (phase 3)	Q2W	Colchicine (1.2 mg Q_AM_, 0.6 mg Q_PM_)	Cardiac arrest	Yes
4	Pegloticase monotherapy (phase 3)	Monthly	Colchicine (0.6 mg BID)	TIA	Yes
5	Pegloticase + MTX (MIRROR RCT)	Q2W	Ibuprofen (200 mg QD)	Cardiac arrest	Yes

a Pegloticase 8-mg infusion; 15 mg/week oral MTX.

BID: twice daily; CHF: congestive heart failure; CV: cardiovascular; DVT: deep vein thrombosis; MI: myocardial infarction; MIRROR: MTX to increase response rates in patients with uncontrolled gout receiving pegloticase; Q2W: every 2 weeks; Q_AM_: every morning; Q_PM_: every night; QD: once daily; QOD: every other day; RCT: randomized controlled trial; TE: thromboembolic; TIA: transient ischemic attack.

A total of 3/244 (1.2%) patients who received on-label (Q2W) pegloticase dosing experienced a single CV/TE event (pegloticase monotherapy [phase 3 trials]: 2/134 patients [1.5%]; pegloticase + MTX [MIRROR RCT]: 1/96 patients [1.0%]). The calculated incidence of CV/TE events with Q2W dosing was 35.4 events/1000 person-years.

## Discussion

In theory, gout is the most understood and manageable of the rheumatic diseases [22]. However, 180 000 patients in the US are estimated to have chronic refractory gout, defined as oral ULT inefficacy or intolerance, persistent hyperuricemia and ongoing gout symptoms [21, 23, 24]. Evidence suggests that patients with uncontrolled gout have a higher gout burden, particularly with respect to frequent and/or prolonged flares, tophus presence, chronic tender and/or swollen joints and an overall decreased quality of life [6]. Pegloticase can effectively and intensively lower SU levels in these patients, with successful treatment resulting in sustained reduction of SU levels below 6 mg/dl [16, 18, 19]. Accordingly, monosodium urate crystal deposits subsequently deplete, and the incidence of gout flare and number of gout-affected joints decrease, positively impacting patient quality of life [16, 17, 20, 25–27].

Patients with gout have been shown to be at an increased risk of TE and CV events in the 30 and 120 days, respectively, following an acute flare [12, 13]. As with all ULTs, initiation of pegloticase increases the risk of patients experiencing a flare. No CV-related safety signals were previously identified in clinical trials of pegloticase, but given these findings, we further investigated the incidence of CV/TE events in patients treated with pegloticase by pooling existing clinical trial data in this *post hoc* analysis. In total, 1.5% of patients who initiated pegloticase experienced a CV/TE event during treatment. In patients receiving biweekly (on-label) pegloticase, 1.2% of patients experienced a CV/TE event. Consistent with prior trials, all CV/TE events occurred within 120 days of gout flare onset [12, 13]. The overall CV/TE event incidence with pegloticase initiation in the clinical trial setting was 35.4 events/1000 person-years. This rate was similar to that of the general gout population (31.7 events/1000 person-years) [10]. Of note, patients in the current analysis who experienced ≥1 treatment-emergent flares had a higher flare rate prior to treatment than those who did not (10.9 ± 13.6 vs 5.3 ± 5.7 flares in the prior 12 months).

When beginning treatment to lower SU levels, patient education is needed to explain that while initiation of any ULT can result in gout flare and that there is a higher risk of CV/TE for several months post-flare, proper SU control greatly reduces the number of flares over the long term [12, 13, 17]. However, the current study suggests that there is not an increased risk of CV/TE events following pegloticase initiation. When considering various treatment approaches for gout, decreasing and preventing flares over the long term is important for reducing gout-related pain, improving quality of life and potentially preserving patient overall health.

This *post hoc* analysis had several strengths, including the relatively large sample size and the fact that the data were drawn from clinical trials, which have a high capture rate of adverse events. Limitations of this analysis included its retrospective nature and those from the original trials. First, trial sites were located in the US, Canada and Mexico, limiting the current findings to a North American population [16, 18, 19]. Second, gout flares were identified via patient interview in the MIRROR trials and were self-reported by patients in the phase 3 trials and not independently verified, potentially leading to over-reporting of flares and differences between the recorded and actual date of flare onset [16]. This *post hoc* analysis had inherent limitations, including treatment heterogeneity (pegloticase dosing schedule and MTX co-therapy use) and a lack of statistical power for hypothesis testing. Though this analysis only examined CV/TE events during the first 6 months of pegloticase therapy, it should be noted that many patients receive longer courses of therapy. Data following discontinuation from the trials were not included in the current analysis, and any CV/TE events that occurred following pegloticase discontinuation were not captured. Given that few CV/TE events occurred across the trials, it was not possible to determine the influence of MTX co-therapy on CV risk.

In conclusion, treatment with pegloticase did not increase the frequency of CV/TE events, and the overall incidence of CV/TE events reported in this *post hoc* analysis was similar to that in the general gout population. Further, all identified CV/TE events occurred within 120 days after gout flare onset, a window of higher risk determined by a prior study [13]. Given that gout flare occurrence is lower in patients who maintain SU levels <6 mg/dl, ULT use in a treat-to-target SU approach not only is an important component of gout management but could play a role in maintaining long-term health in patients with gout.

## Data Availability

Qualified researchers may request data from Amgen clinical studies. Complete details are available at the following: https://www.amgen.com/science/clinical-trials/clinical-data-transparency-practices/clinical-trial-data-sharing-request.
